# Development of a Modular miRNA-Responsive Biosensor for Organ-Specific Evaluation of Liver Injury

**DOI:** 10.3390/bios14090450

**Published:** 2024-09-20

**Authors:** Xinxin Zhang, Tingting Wang, Xiangqing Fan, Meixia Wang, Zhixi Duan, Fang He, Hong-Hui Wang, Zhihong Li

**Affiliations:** 1 College of Biology, Hunan University, No. 27 Tianma Road, Yuelu District, Changsha 410082, China; zxx120203@hnu.edu.cn (X.Z.); wangtingting@hnu.edu.cn (T.W.); fxqing@hnu.edu.cn (X.F.); wangmeixia@hnu.edu.cn (M.W.); fanghe@hnu.edu.cn (F.H.); 2 Department of Emergency Medicine, The Second Xiangya Hospital of Central South University, Changsha 410011, China; xiangyadzx@csu.edu.cn; 3 Department of Trauma Center, The Second Xiangya Hospital of Central South University, Changsha 410011, China; 4 FuRong Laboratory, Changsha 410078, China; 5 Department of Orthopaedics, The Second Xiangya Hospital of Central South University, Changsha 410011, China

**Keywords:** biomarkers, disease-associated microRNA, DNA nanotechnology, liver injury, fluorescence probe, organ-specific biosensing

## Abstract

MicroRNAs (miRNAs) are increasingly being considered essential diagnostic biomarkers and therapeutic targets for multiple diseases. In recent years, researchers have emphasized the need to develop probes that can harness extracellular miRNAs as input signals for disease diagnostics. In this study, we introduce a novel miRNA-responsive biosensor (miR-RBS) designed to achieve highly sensitive and specific detection of miRNAs, with a particular focus on targeted organ-specific visualization. The miR-RBS employs a Y-structured triple-stranded DNA probe (Y-TSDP) that exhibits a fluorescence-quenched state under normal physiological conditions. The probe switches to an activated state with fluorescence signals in the presence of high miRNA concentrations, enabling rapid and accurate disease reporting. Moreover, the miR-RBS probe had a modular design, with a fluorescence-labeled strand equipped with a functional module that facilitates specific binding to organs that express high levels of the target receptors. This allowed the customization of miRNA detection and cell targeting using aptameric anchors. In a drug-induced liver injury model, the results demonstrate that the miR-RBS probe effectively visualized miR-122 levels, suggesting it has good potential for disease diagnosis and organ-specific imaging. Together, this innovative biosensor provides a versatile tool for the early detection and monitoring of diseases through miRNA-based biomarkers.

## 1. Introduction

Effective disease diagnosis depends on a deep understanding of the complex changes in the body. Traditional diagnostic methods such as blood tests, imaging, biopsies, endoscopy, and genetic testing are associated with high rates of false positives and require further confirmation through reliable biomarkers [1]. Biomarkers associated with various biological processes, pathogenic changes, and treatment responses are often applied in research and clinical practice to facilitate early diagnosis and non-invasive disease detection. However, traditional biomarkers like alanine transaminase (ALT), aspartate transaminase (AST), alkaline phosphatase (ALP), and bilirubin levels have several limitations in terms of specificity and sensitivity, which limit their application in early liver disease detection [2]. Recently, microRNAs (miRNAs) have emerged as promising biomarkers due to their high fidelity, easy accessibility, and high cellular and tissue specificity [3]. The evidence in the literature indicates that abnormal miRNA expression contributes to the occurrence of various human diseases [4,5,6]. For example, miR-122 is one of the most abundant miRNAs in the liver, accounting for 70% and 52% of the total liver miRNA pool in adult mice and humans, respectively [7,8,9]. During liver injury, miR-122 is secreted by necrotic liver cells, significantly increasing its circulating levels by 100 to 10,000 times compared to normal liver [10,11,12,13]. The Food and Drug Administration (FDA) has approved miR-122 as an exploratory biomarker for drug-induced liver injury in clinical trials. This suggests that miRNAs have great potential to facilitate non-invasive diagnosis, disease progression prediction, treatment guidance, and the evaluation of responses to therapies.

Notably, rapid and robust multiplex miRNA detection methods are crucial in biological research and clinical diagnostics. Traditional miRNA detection techniques, such as Northern blot [14,15,16], microarrays [17,18], and qPCR [19,20], are limited by high costs, high time consumption, and low sensitivity [21]. Several novel detection methods have emerged to address these challenges such as nanomaterial-based miRNA detection. Due to their high surface area, excellent conductivity, and remarkable chemical stability, nanomaterials have become powerful tools for enhancing the performance of traditional detection methods. For example, gold nanoparticles (AuNPs) [22], silver nanoclusters (AgNCs), copper nanoparticles (CuNPs) [23], and DNA nanomaterials [24] have all been widely utilized to develop more sensitive tools for miRNA detection. In addition, miRNA detection methods based on nucleic acid amplification technologies have also been developed. In recent years, various nucleic acid amplification techniques have emerged, including rolling circle amplification (RCA) [25,26,27], dual-specific nuclease (DSN)-based amplification [28], loop-mediated isothermal amplification (LAMP), strand displacement amplification (SDA) [29], and various enzyme-free amplification methods. These methods can be harnessed to detect low levels of miRNA in tissues and cells, and the nucleic acid products generated from the above amplification techniques can be detected using various signal readout methods, such as electrochemical [30], fluorescence [31], chemiluminescence [32], and colorimetric assays. Among these, fluorescence-based detection methods are particularly favored due to their high sensitivity, strong selectivity, and real-time detection capabilities, making them widely applicable in biosensing. In recent years, many researchers have tried to apply these miRNA detection methods to disease detection. For example, Lee et al. [33] developed an innovative detection system for multiple cancer biomarker miRNAs by integrating transcription-mediated isothermal amplification with tetrahedral DNA nanostructures (TDNs). However, these methods cannot provide information on the spatial distribution of miRNA in target tissues within the organism. 

The development of nucleic acid aptamers has offered a new approach to addressing these localization challenges. Aptamers are structured single-stranded oligonucleotides capable of binding to various targets with high affinity [34,35]. For example, the Sando research group reported a nucleic acid aptamer that mimics the basic fibroblast growth factor (bFGF) function, which can specifically bind to the fibroblast growth factor receptor (FGFR) on the cell membrane [36]. Additionally, they developed an aptamer that mimics hepatocyte growth factor (HGF) and binds to the MET receptor, a receptor tyrosine kinase (RTK) [37]. The targeting properties of nucleic acid aptamers can help us to explore the location information of miRNA in the organism to achieve the precise location of organs or disease sites.

**Scheme 1:**
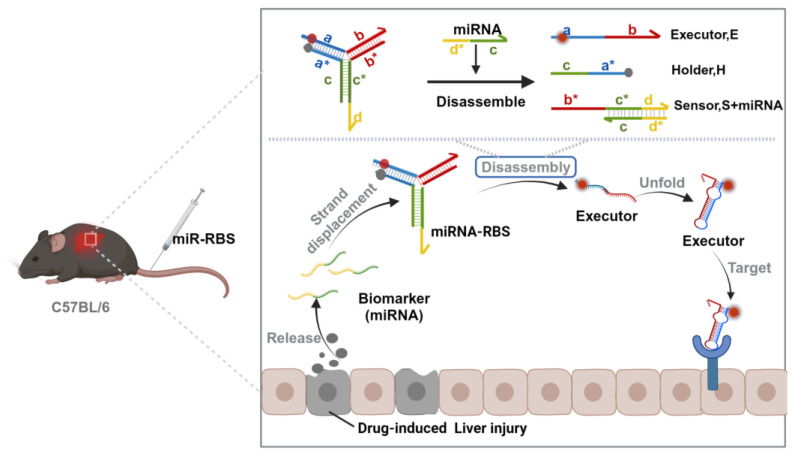
A schematic diagram of the miRNA-responsive biosensor (miR-RBS) that utilizes a Y-structured triple-stranded DNA probe (Y-TSDP) to precisely localize liver injury sites. (The letters with * are the complementary strands of the corresponding letters without *). The Y-TSDP remains in a fluorescence-quenched state under normal conditions. Following the detection of specific miRNA biomarkers, such as miR-122, released during drug-induced liver injury, the probe undergoes strand displacement, leading to the disassembly of the Y-structure and activation of the fluorescence signal. The activated executor module then targets specific receptors overexpressed on liver cells, enabling organ-specific imaging and accurate disease diagnosis. The figure was created with BioRender.com.

In this study, we leveraged the DNA nanotechnology to design a novel miRNA-responsive biosensor (miR-RBS) for miRNA detection and organ-specific imaging. This biosensor comprised a Y-structured triple-stranded DNA probe (Y-TSDP) that remains fluorescence-quenched under basal conditions and activates upon miRNA binding through a rapid displacement reaction (Scheme 1). The fluorescent-labeled chain of the miR-RBS contains a targeting module that specifically to binds to overexpressed receptors in the target organs, enabling precise organ-specific imaging. The modular design allows the customized detection of different miRNAs and targeting of specific cells, making it a versatile tool for the diagnosis of various diseases.

## 2. Materials and Methods

### 2.1. Chemicals and Reagents

The oligonucleotide sequences used in this study were synthesized by Shanghai Sangon Biotech Company and purified using high-performance liquid chromatography (see Tables S1–S3. All oligonucleotides were dissolved in phosphate-buffered saline (PBS) at pH 7.4 and adjusted to a concentration of 10 μM, finally stored at −20 °C for later use. Dulbecco’s Modified Eagle Medium (DMEM) medium was purchased from BasalMedia Biotechnology (Shanghai, China). Fetal bovine serum (FBS) was purchased from Bio-Channel (Nanjing, China).

### 2.2. Instruments

A SynergyTM Mx Multi-Mode Microplate Reader (BioTek, Winooski, VT, USA) was used to perform fluorescence intensity measurements. Confocal fluorescence imaging was conducted with a Confocal Laser Scanning Microscope (Leica, Wetzlar, Germany). In vivo imaging of animals was conducted using a multi-modal small animal imaging system (PerkinElmer, Waltham, MA, USA) to obtain live animal images.

### 2.3. Polyacrylamide Gel Electrophoresis (PAGE)

To analyze the assembly of the designed DNA nanostructures and the disassembly triggered by released miR-122, we performed agarose gel electrophoresis. The reaction protocol was as follows: the sensor module (S), executing module (E), and holding module (H) were heated at 37 °C for 2 h to allow assembly, followed by the addition of miR-122 and further incubation for 30 min. At the end of the reaction, the samples were loaded onto a 12% native PAGE and run at 120 V in 1 × Tris-borate-EDTA (TBE) buffer (89 mM tris (hydroxymethyl) aminomethane, 2 mM ethylenediaminetetraacetic acid, and 89 mM boric acid, pH 8.0) for 50 min. The gel was run with the Gel-Red for 20 min and scanned using an automated gel imaging system.

### 2.4. Fluorescence Measurement 

The disassembly of the miR-122-RBS probe triggered by miR-122 was monitored in real time by detecting the fluorescence in 100 μL PBS buffer (pH = 7.4). The buffer contained assemblies of different concentrations (S, E, and H modules annealed overnight at 37 °C) at 37 °C. Fluorescence intensity was monitored using a fluorescence spectrometer at 649~670 nm to characterize the reaction process.

### 2.5. Cell Culture

The cell lines were cultured in a 5% CO_2_ incubator (Thermo Fisher, Waltham, MA, USA) at 37 °C. Buffalo Rat Liver (BRL) cells and Mouse embryonic cells (NIH-3T3) cells were cultured in DMEM supplemented with 10% FBS and 1% penicillin and streptomycin. 

### 2.6. Cell Imaging 

Cells are cultivated in 35 mm confocal dishes with complete medium for 24 h at 5% CO_2_ and 37 °C. BRL cells were first washed and incubated with miR-122-RBS-MET (200 nM) and miR-122 (200 nM) in 1 mL buffer at 4 °C for 30 min. Similarly, the NIH-3T3 cells were washed and incubated with miR-122-RBS-FGFR (200 nM) and miR-122 (200 nM) in 1 mL buffer at 4 °C for 30 min. Subsequently, Confocal fluorescence imaging was conducted using a Confocal Laser Scanning Microscope (Leica, Wetzlar, Germany) with a 63 × oil immersion objective. The recordings were performed using the Sulfo-Cyanine5 Cy5 channel (at the excitation wavelength of 640 nm and emission wavelength of 670 nm). The fluorescence intensity generated on the cell surface was analyzed and quantified using the Image J v1.53a software program.

### 2.7. Animal Studies

All experiments were performed in accordance with the “Regulations on the Administration of Experimental Animals by the Ministry of Science and Technology of the People’s Republic of China”. All procedures involving animal experiments were reviewed and approved by the Hunan University Committee on Experimental Animal Ethics (HNUBIO202302001) and the Hunan Provincial Center for Experimental Animals Animal Experiment Ethics Committee [SYXK (Xiang) 2018-0006]. C57BL/6 adult male mice (8–9 weeks old) were purchased from Hunan Slac Jingda Experimental Animal Co., Ltd. (Changsha, China), and housed under virus-free and pathogen-free conditions. The mice were reared in cages with up to 5 mice per cage and provided food and water ad libitum. The room was adjusted to a 12 h light/12 h dark cycle.

### 2.8. Drug-Induced Acute Liver Injury Model

A mouse model of drug-induced acute liver injury was established following a previous study [38]. Twenty adult male C57BL/6 mice (8–9 weeks old) were randomly divided into a control group and an Acetaminophen (APAP) injury group, with 10 mice in each group. To deplete the glutathione levels in hepatocytes and create comparable conditions for APAP metabolism, both groups of mice were fasted for 12 h prior to treatment, with free access to water. Subsequently, the APAP injury group received an intraperitoneal injection of APAP at a dose of 400 mg/kg body weight, while the control group received an equivalent volume of PBS solution via intraperitoneal injection. Twelve hours after induction, 5 mice from each group were randomly selected to receive a tail vein injection of miR-122-RBS-MET, while the remaining 5 mice in each group received a tail vein injection of PBS. Animal imaging was performed 1 h after the tail vein injection. The performance of miR-122-RBS-MET in vivo was validated through animal experiments to investigate its applicability to target sites of drug-induced acute liver injury. 

### 2.9. Statistics

The data are presented as mean ± standard deviation (s.d.). The difference between the two groups was compared with a two-tailed Student’s *t*-test. The statistical significance was calculated using Student’s *t*-test. NS: not significant; * *p* < 0.05, ** *p* < 0.01, and *** *p* < 0.001 indicate significant levels. All statistical analyses were performed using GraphPad Prism v.9.

## 3. Results

### 3.1. Design and Working Model of miR-RBS

We propose a molecular engineering strategy for designing a modular miRNA-responsive nano-probe that selectively responds to disease-related miRNA biomarkers to target specific organs and achieve precise disease site targeting. This method leverages the dynamic DNA nanotechnology, enabling the construction of a programmable and tunable biosensing system [39,40]. Using this method, we designed a Y-structured three-stranded DNA nanodevice responsive to miRNA, composed of three modules: a sensor module (S) responsive to miRNA, an executing module (E) targeting specific diseased cells, and a holding module (H) (Scheme 1). In the absence of miRNA, the targeting chain of the E module is blocked by the binding domains (b* from the S module and a* from the H module) through complementary base pairing to form a three-stranded DNA complex (Figure S1). 

In the absence of microRNA input, the effector module is inhibited, preventing it from targeting specific cells. However, when a specific microRNA binds to the sensor module, the inhibitory region on the effector module is released, allowing the Y-shaped DNA complex to disassemble and release the targeting chain. The targeting chain targets specific cell receptors at the disease site. The designed blocked region allows the precise control of the miRNA responsiveness, sensitivity, and reaction kinetics of the miR-RBS. The designed miR-RBS platform exhibited high sensitivity, specificity, and tunability, facilitating customization of the probe to meet specific requirements.

**Figure 1:**
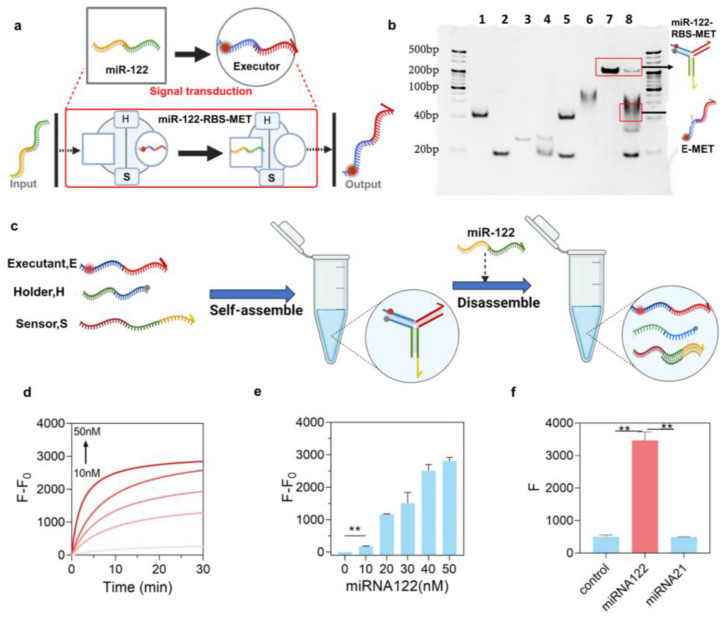
Characterization of the miR-RBS. (**a**) Schematic representation of the miR-RBS design. The Y-structured triple-stranded DNA probe (Y-TSDP) consists of three modules: the sensor module (S), the holding module (H), and the executor module (E). In the presence of miR-122, the probe undergoes strand displacement, resulting in the disassembly of the Y-structure and activation of the executor module. The figure was created with BioRender.com. (**b**) Gel electrophoresis analysis of the miR-RBS assembly and disassembly. Lane 1: executor module (E). Lane 2: holding module (H). Lane 3: sensor module (S). Lane 4: assembled executor module and holding module (E + H). Lane 5: assembled executor module and sensor module (E + S). Lane 6: assembled holding module and sensor module (H + S). Lane 7: assembled Y-TSDP (E + H + S). Lane 8: miR-122-RBS-MET disassembled by miR-122. (**c**) Schematic of the self-assembly and disassembly process of the Y-structured triple-stranded DNA probe (Y-TSDP) upon interaction with miR-122. The figure was created with BioRender.com. (**d**) Real-time fluorescence response of the miR-RBS to varying concentrations of miR-122 (0–50 nM, The darker the color of the curve, the higher the concentration), illustrating a rapid increase in fluorescence signal upon miR-122 binding. (**e**) Quantification of the fluorescence intensity (F/F0) at different miR-122 concentrations, demonstrating the sensitivity of the miR-RBS. (**f**) Specificity test of the miR-RBS. Fluorescence intensity was measured in the presence of miR-122 and miR-21, showing a significant response to miR-122 compared to the control and miR-21 groups. Data are presented as the mean ± S.D. (n = 3). Error bars represent standard deviation, and ** indicates *p* < 0.01. (Unpaired *t*-test, two-tailed *p* value, n = 3).

To validate the performance of the probe, we established a mouse model of the miR-122/liver injury. miR-122 is an extracellular biomarker associated with various liver diseases, such as non-alcoholic fatty liver disease (NAFLD) and chronic hepatitis [41]. The responsiveness of miR-RBS was combined with the endogenous receptor required for targeting to achieve precise localization of specific cells. To explore the feasibility of this approach, we chose the MET receptor, a typical RTK that binds to HGF and regulates liver regeneration after injury [42]. Based on the target miR-122 sequence and MET-specific aptamer sequence, we designed and constructed a miR-122-responsive RBS (miR122-RBS-MET) (Figure 1a and Figure S1 and Table S1). 

To verify the feasibility of miR-RBS, the assembly and conformational disassembly of miR122-RBS-MET were examined using nucleic acid gel electrophoresis. The result confirmed the successful assembly of the E, H, and S modules (Figure 1b, lane 7) and responsive conformational disassembly following miR-122 input (Figure 1b, lane 8). To further evaluate the responsiveness of miR-RBS, we designed a quencher-fluorophore “light-up” system, with Cy5 modification at the 12th position of the T base in the E chain and Black Hole Quencher 1 (BHQ1) modification at the corresponding position in the H chain. In the absence of the miR-122 input, the structure remains stable, and the Cy5 fluorescence on miR-122-RBS-MET is quenched by BHQ1 without generating fluorescence. Following the introduction of miR-122, the miR-122-RBS-MET-responsive conformation, with the quencher conjugated strand on the H module separated from the E module, promotes the recovery of the fluorescence signal of the released E module (Figure S2). Hence, this transformation converted the miR-122 signal at the input end to a fluorescence signal at the output end (Figure 1c). 

Subsequently, an in vitro fluorescence analysis was conducted to determine whether a fluorescence signal was generated in the absence of miR-122. The results showed no signal was produced, indicating non-specific leakage in the reaction. On the contrary, the presence of the miR-122 target significantly increased the Cy5 fluorescence signal by 24-fold, and as the concentration of miR-122 increased, the Cy5 fluorescence intensity significantly increased, with the reaction kinetics exhibiting a positive correlation with the level of miR-122 (Figure 1d). A quantitative analysis indicated low concentrations of 10 nM and 20 nM, much lower than the pathological concentration range of miR-122 (sub-micromolar level) and indicative of liver injury [38,43,44]. miR122-RBS-MET detected miR-122 with high sensitivity, which was significantly different from the control group (Figure 1e). In a subsequent analysis, the target specificity of miR122-RBS-MET was explored using another small endogenous non-coding RNA, miR-21. It was observed that the fluorescence intensity after the addition of miR-21 was significantly lower compared with that of the miR-122 group (Figure 1f). These data confirmed that miR122-RBS-MET exhibited sequence-specific selectivity, distinguishing key regions of miR-122 (c* and d) from other miRNAs. Together, these findings indicate that the designed miR-RBS could detect the target miRNA with high sensitivity and specificity.

### 3.2. The Tunable Performance of miR-RBS

**Figure 2:**
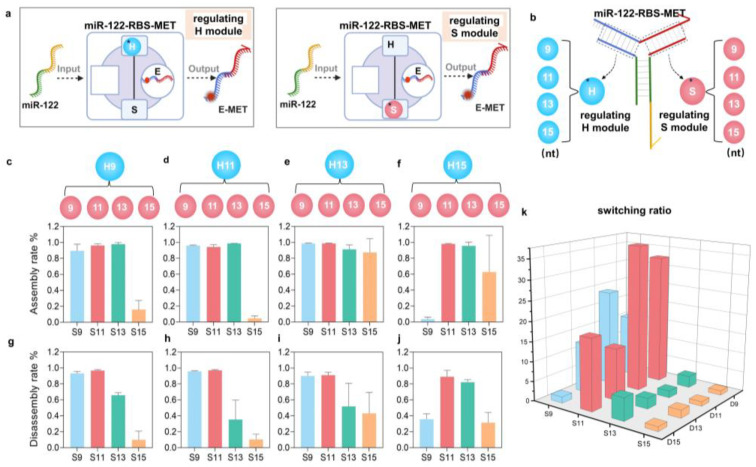
Engineering miR-RBS with adjustable sensitivity and specificity. (**a**) Schematic representation of the miR-122-RBS-MET design with adjustable modules. The regulating H module and regulating S module are engineered to fine-tune the response of the biosensor. The figure was created with BioRender.com. (**b**) Detailed illustration of the adjustable regions within the miR-RBS-MET, showing the positions of the regulating H module (9–15 nt) and the regulating S module (9–15 nt). (**c**–**f**) Assembly rates of the miR-RBS-MET with different lengths of the regulating H module (9, 11, 13, 15 nt) and regulating S module (9, 11, 13, 15 nt). Each bar graph indicates the efficiency of assembly at varying combinations of module lengths, demonstrating high assembly efficiency across various configurations. (**g**–**j**) Disassembly rates of the miR-RBS-MET with different lengths of the regulating H module (9, 11, 13, 15 nt) and regulating S module (9, 11, 13, 15 nt). Each bar graph shows the efficiency of disassembly upon miR-122 binding, indicating the sensor’s responsiveness and tunability. (**k**) Switching ratios of the miR-RBS-MET, calculated as the degree of deconstruction after miR-122 input divided by the degree of deconstruction without input. The 3D bar graph displays the adjustable sensitivity of the biosensor, with different combinations of regulating H and S module lengths providing a wide range of switching ratios.

Considering the varying concentrations of miRNA in different diseases and normal states, the designed miR-RBS should be capable of adjusting to specific conditions. Next, we investigated whether the dynamic range of the miR122-RBS-MET-responsive miRNA conformation release module can be controlled through molecular engineering to meet varying sensitivity needs. Specifically, we modified the closed regions of the E module and investigated the adjustability of miR-RBS. The region **a** of the H module to the closed region of the E module was set to 9 nt, 11 nt, 13 nt, and 15 nt, and the region **b** of the S module to the closed region of the E module was also set to four ranges of 9 nt, 11 nt, 13 nt, and 15 nt (Figure 2a,b and Table S2). Subsequently, the closed region of the H module was fixed to the E module and the closed region of the S module was changed. This allowed us to calculate the assembly rate of the design and the disassembly rate corresponding to miR-122 by running a nucleic acid gel. The results showed that the assembly rate of most assemblies was relatively high in the range of 9–15 nt for region **a** and 9–13 nt for region **b**, with the majority exceeding 90% (Figure 2c–f and Figure S3). Moreover, when the number of complementary bases in the closed region is between 9 and 15 nt, the efficiency of the response disassembly of miR122-RBS-MET varies, exhibiting different sensitivity levels (Figure 2g–j and Figure S4). Finally, the switch ratio results were calculated using the gray value of PAGE. It was observed that the miR-122-RBS-MET achieved different switch effects by adjusting the length of the closed region, with an adjustable switch ratio ranging from 1.6:1 to 37:1 (Figure 2k). Therefore, the proposed miR-RBS platform has many tunable regions, allowing the customization of specific response sensitivities.

### 3.3. Modularity of miR-RBS for Cell-Specific miRNA Visualization

**Figure 3:**
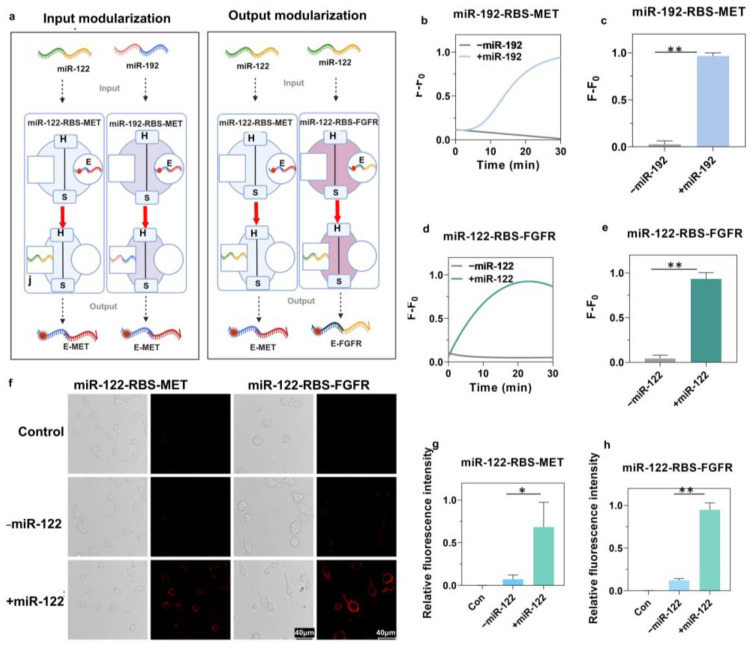
Modular engineering and validation of the miR-RBS for specific miRNA detection and cell-specific targeting. (**a**) Schematic representation of input and output modularization of the miR-RBS. The input modularization involves replacing the sensor module (S) to respond to different miRNAs (miR-122 and miR-192). The output modularization involves replacing the executor module (E) to target different receptors (MET and FGFR). The figure was created with BioRender.com. (**b**,**c**) Real-time fluorescence response and quantification of miR-192-RBS-MET following the addition of miR-192, demonstrating a significant increase in fluorescence signal, and hence, successful detection. Data are presented as the mean ± S.D. (n = 3). Error bars represent standard deviation, and * indicates *p* < 0.1. (Unpaired *t*-test, two-tailed *p* value, n = 3.) (**d**,**e**) Real-time fluorescence response and quantification of miR-122-RBS-FGFR upon addition of miR-122, showing a significant increase in fluorescence signal confirming the specific detection and targeting capability. Data are presented as the mean ± S.D. (n = 3). Error bars represent standard deviation, and * indicates *p* < 0.05. (Unpaired *t*-test, two-tailed *p* value, n = 3.) (**f**) Confocal microscopy images of cells treated with miR-122-RBS-MET and miR-122-RBS-FGFR in the presence or absence of miR-122. The presence of miR-122 induces a significant increase in the fluorescence signal on the cell surface, confirming successful targeting of the corresponding receptors (MET and FGFR). (**g**,**h**) Quantification of relative fluorescence intensity in cells treated with miR-122-RBS-MET and miR-122-RBS-FGFR, showing significant differences between control, miR-122, and non-miR-122 treated groups. Data are represented as mean ± S.D. (n = 3). Error bars represent standard deviation, and * indicates *p* < 0.05, ** indicates *p* < 0.01. (Unpaired *t*-test, two-tailed *p* value, n = 3).

The designed miR-RBS contains a modular feature that enables the customization of the device by replacing the sensor module with different miRNA inputs or the execution module with different outputs. To test the performance, miR-192 was selected because it was previously found to be elevated in diabetic nephropathy [45] and is one of the key renal miRNA markers. The response region of the S module was replaced with the miR-192 complementary sequence to form miR-192-RBS-MET (Figure 3a). An analysis of the response kinetics indicated that the fluorescence intensity was significantly increased when miR-192 was introduced at the input end of the miR-192-RBS-MET system compared to the control group (Figure 3b,c and Figure S5). 

In further experiments, we exchanged the output end of the functional module, switching the original targeting MET receptor to targeting FGFR, which is crucial for embryonic development and tissue homeostasis [46]. Moreover, we replaced the E module with a specific adapter sequence for targeting FGFR, and we then designed and assembled the miR-122-RBS-FGFR (Figure 3a and Figure S6). This led to a significant increase in the fluorescence intensity in the miR-122-RBS-FGFR system in the presence of miR-122, as demonstrated by results of the response kinetics (Figure 3d,e). To further confirm whether miR-RBS can achieve specific cellular localization in response to miR-122 conformational changes, confocal fluorescence microscopy was conducted which revealed significant fluorescence on the surfaces of BRL cells and NIH-3T3 cells in the presence of miR-122, while no fluorescence was detected on the cell surfaces in the absence of miR-122 (Figure 3f). The quantitative results demonstrated a significant increase in fluorescence intensity in the miR-122-treated group compared to the group without miR-122, with fold changes of 8.49 in BRL cells and 7.72 in NIH-3T3 cells (Figure 3g,h). These findings indicated that miR-122-RBS-MET and miR-122-RBS-FGFR effectively responded to miR-122, enabling the precise targeting of specific cell surfaces with fluorescently labeled MET and FGFR aptamer chains. These results highlighted that the miR-RBS system could be customized at both the input and output ends, enabling selective targeting to the desired cells and receptors.

### 3.4. Organ-Specific Visualization of Disease-Associated miRNA

**Figure 4:**
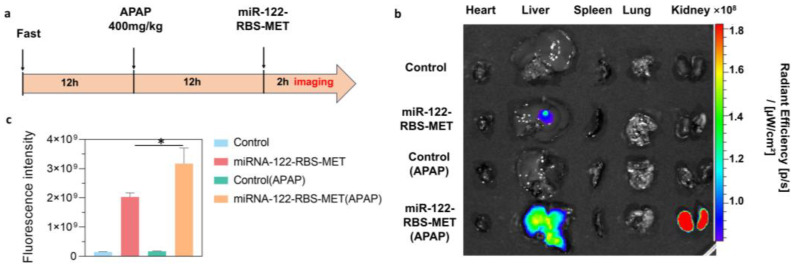
Organ-specific visualization of miRNA associated with liver injury. (**a**) Experimental timeline showing the establishment of an acute liver injury model in mice. Briefly, mice were fasted for 12 h, and then intraperitoneally injected with APAP (400 mg/kg) to induce liver injury. After another 12 h, mice were intravenously injected with either PBS or miR-122-RBS-MET (10 μM, 200 μL). Organ imaging was performed 2 h post-injection. (**b**) Representative fluorescent images of organs (heart, liver, spleen, lung, kidney) from the indicated experimental groups: Control, miR-122-RBS-MET, Control (APAP), and miR-122-RBS-MET (APAP). Significant fluorescence was detected in the liver of the miR-122-RBS-MET (APAP) group, indicating specific detection of miR-122. Fluorescence was also observed in the kidneys, suggesting that the biosensor was processed after filtration. (**c**) Quantification of the fluorescence intensity in the liver, displaying significantly higher intensity in the miR-122-RBS-MET (APAP) group than in the other groups. Data are presented as the mean ± S.D. (n = 3). Error bars represent standard deviation, and * indicates *p* < 0.05. (Unpaired *t*-test, two-tailed *p* value, n = 3).

To further investigate whether miR-122-RBS-MET could respond to the liver injury biomarker miR-122 in vivo, we first replaced the Cy5 fluorescence of module E with the more penetrating Cy7 fluorescence, similarly to replacing the quenching groups of module H with BHQ3 (Table S3). Next, we established a mouse model of drug-induced liver injury (DILI) (Figure 4a). Common drugs that cause DILI include APAP, herbal medicines, statins, and anti-tuberculosis drugs. The clinical manifestations of DILI vary depending on the causative drug, among which APAP-induced acute liver injury is the most extensively studied [47]. Therefore, we established a mouse model of APAP-induced acute liver injury to examine the performance of miR-122-RBS-MET. We injected the labeled miR-122-RBS-MET into APAP mice and rested for 1 h under the small animal live imaging system (λ ex = 750 nm, λ em = 773 nm). However, due to the limited penetration of Cy 7 fluorescence, we failed to detect fluorescence in vivo, and we then euthanized the mice to obtain heart, liver, spleen, lung, and kidney after our miR-122-RBS-MET 2 h, and again under the imaging system (λ ex = 750 nm, λ em = 773 nm) (Figure 4b). Imaging of mice organs (hearts, livers, spleens, lungs, and kidneys) showed that the signal of miR-122-RBS-MET in healthy mice was mainly in a quenched state, indicating that the miR-122-RBS device structure remained intact under physiological conditions. However, in the miR122-RBS-MET (APAP) group mice, significant fluorescence was detected in the liver region with the signal-to-background ratio increasing to 1.96 times (Figure 4c), indicating that the biosensor could specifically detect disease-related miRNAs, effectively distinguishing between healthy and injured organisms. Moreover, significant fluorescence was seen in the kidney region, which is consistent with the results of Xiao et al. [48] in their study on the distribution and pharmacokinetics of nucleic acid aptamers in vivo, which flow through various organs of the whole body via the blood circulation, and most of them are accumulated in the kidney site. In this study, miR-122-RBS-MET released fluorescent aptamer chains in response to miR-122 deconjugation in the liver, which were targeted to bind to the overexpressed MET receptor on the surface of hepatocytes at the lesion site, and the rest of the aptamers that were not bound to the aptamer were accumulated in the kidneys through the bloodstream and cleared, so that a large amount of fluorescence could be observed in the kidney site as well.

## 4. Discussion

In this study, we have developed a new modular biosensor for extracellular miRNA sensing and response. The probe could accurately target receptors on the cell surface enabling efficient disease discrimination at the organ level. The modular miR-RBS allows easy customization of miRNA-responsive input and target receptor output based on user requirements, with adjustable sensitivity. To our knowledge, there are an increasing number of biosensors developed for miRNA detection based on DNA nanotechnology. For example, Xian Chen et al. [49] designed a switchable ratio fluorescence biosensor (SCRF biosensor) that achieves the highly sensitive and rapid quantitative detection of miRNA through the conversion of DNA switch structures and the ratio of fluorescence signals. Ruiying Yang et al. [50] designed a dual-mode biosensor based on a 3D DNA Walker strategy, which realizes the ultra-sensitive detection of miRNA-224 through signal enhancement and quenching. These sensors have potential practical applications in bioanalysis and early disease diagnosis. The experimental results demonstrated that the miR-RBS was effective in disease detection. Currently, in vivo organ imaging is challenging due to the lack of suitable fluorescent moieties that can efficiently penetrate tissues. Therefore, based on our findings, we hope that our probe may improve in vivo animal disease detection.

The miR-RBS provides a rapid, sensitive, customizable, non-invasive, and non-intrusive method for disease detection. The designed miR-122-RBS-MET not only detects the disease state of drug-induced liver injury but also other liver-related diseases using miR-122, making it a suitable diagnostic biomarker for diseases, such as non-alcoholic fatty liver disease (NAFLD) [51], chronic hepatitis B or C [52,53], and hepatocellular carcinoma (HCC) [54]. This broadens the diagnosis application of miR-122-RBS-MET in various liver-related diseases. Our miR-RBS provides a platform for the future development of programmable and customizable molecular devices that will facilitate the exploitation of biomarker miRNAs. 

Despite the promising results, there are several challenges that should be acknowledged. Currently, there are no fluorescent moieties that can efficiently penetrate organs to allow in vivo imaging. In future, researchers should develop fluorescent markers with high penetrative power to enhance the in vivo imaging and real-time monitoring of disease states. Another challenge lies in the scalability and implementation of this technology in clinical settings. Further investigations need to test the stability and reproducibility of the miR-RBS in diverse biological environments. Additionally, the integration of this biosensor with existing diagnostic platforms in a cost-effective manner needs to be explored to promote its widespread adoption. 

In summary, the designed miR-RBS provides a versatile platform for early disease detection and monitoring leveraging miRNA-based biomarkers. The customizability of the biosensor for different miRNAs and target cells makes it a powerful tool for various biomedical applications. Therefore, we anticipate that the continuous improvement of DNA nanotechnology and fluorescent probes through further research will expand the diagnostic and therapeutic capabilities of the miR-RBS.

## Data Availability

The data supporting the findings of this study are available from the corresponding author upon reasonable request.

## Supplementary Materials

The following supporting information can be downloaded at: https://www.mdpi.com/article/10.3390/bios14090450/s1, Table S1: Oligonucleotide sequences for characterization of miR-RBS; Table S2: Oligonucleotide sequences for modularization of miR-RBS; Table S3: Oligonucleotide sequences for functional validation in in vivo imaging; Figure S1. The schematic representation of miR-122-RBS-MET with detailed sequences; Figure S2. Linear fitting between the initial fluorescence intensity (F.I.) and the concentration of the Executor module; Figure S3. Secondary structure prediction of miR-RBS assembled in altered regulatory regions using the NUPACK software (version 4.0); Figure S4. Validation of the assembly of regulatable miR-RBS and disassembly in response to miR-122 using PAGE; Figure S5. Secondary structure prediction of miR-RBS modularity using the NUPACK software; Figure S6. Validation of the miR-RBS assembly after modularization and response disassembly.
