# Supplementary material for: Development of a Modular miRNA-Responsive Biosensor for Organ-Specific Evaluation of Liver Injury
Source: Biosensors (Basel). 2024 Sep 20;14(9):450. doi: 10.3390/bios14090450 (PMC11430419; doi:10.3390/bios14090450)
Supplement: Supplementary file 1 [file biosensors-14-00450-s001.zip › biosensors-3122140-supplementary.pdf]

Supporting Information

Title: Development of a Modular miRNA-Responsive Biosensor for Organ-specific Evaluation of Liver Injury

Xinxin Zhang[1,+],Tingting Wang[1, +],Xiangqing Fan[1], Meixia Wang[1], Zhixi Duan [2, 3, 4], Fang He[1], Hong-Hui Wang[1], Zhihong Li [3, 4, 5]

1. College of Biology, Hunan University, 27 Tianma Road, Yuelu District, Changsha, Hunan 410082, China.
2. Department of Emergency Medicine, The Second Xiangya Hospital of Central South University, Changsha, 410011, Hunan, China.
3. Department of Trauma Center, The Second Xiangya Hospital of Central South University, Changsha, 410011, Hunan, China.
4. FuRong Laboratory, Changsha 410078, Hunan, China
5. Department of Orthopaedics, The Second Xiangya Hospital of Central South University, Changsha, 410011, Hunan, China.

* Correspondence:
H.W., wanghonghui@hnu.edu.cn; Tel.: +86-15367809537
Z. L., lizhihong@csu.edu.cn; Tel.: +86- 13975112458

+ These authors contributed equally to this work.

Table S1. Oligonucleotide sequences for characterization of miR-RBS

Primer Name	Sequence (5'---3')
miR-122-RBS-MET-E	ATCAGGCTGGATGGTAGCTCGGTCGGGGTGGGTGGGTTGGCAAGTCTGAT
D-9	TGAGAATGGTGTTTGCCGAGCTAC
H-11	TGAGAATGGTGTTTGCCGAGCTACCA
H-13	TGAGAATGGTGTTTGCCGAGCTACCATC
H-15	TGAGAATGGTGTTTGCCGAGCTACCATCCA
S-9	CCCACCCCGCAAACACCATTGTCACACTCCA
S-11	CACCCACCCCGCAAACACCATTGTCACACTCCA
S-13	CCCACCCACCCCGCAAACACCATTGTCACACTCCA
S-15	AACCCACCCACCCCGCAAACACCATTGTCACACTCCA
miR-122-RBS-MET-E-Cy5	ATCAGGCTGGA/iCy5dT/GGTAGCTCGGTCGGGGTGGGTGGGTTGGCAAGTCTGAT
miR-122-RBS-MET-H-BHQ1	TGAGAATGGTGTTTGCCGAGCTACCA(BHQ1)
miR-122-RBS-MET-S	CACCCACCCCGCAAACACCATTGTCACACTCCA
miR-122	UGGAGUGUGACAAUGGUGUUUG
miR-122-mimic	TGGAGTGTGACAA TGGTGTTTG
miR-21	UAGCUUAUCAGACUGAUGUUGA

Table S2. Oligonucleotide sequences for modularization of miR-RBS

Primer Name	Sequence (5'---3')
miR-122-RBS-MET-E-Cy5	ATCAGGCTGGA/iCy5dT/GGTAGCTCGGTCGGGGTGGGTGGGTTGGCAAGTCTGAT
miR-122-RBS-MET-H-BHQ1	TGAGAATGGTGTTTGCCGAGCTACCA(BHQ1)
miR-122-RBS-MET-S	CACCCACCCCGCAAACACCATTGTCACACTCCA
miR-192-RBS-MET-E-Cy5	ATCAGGCTGGA/iCy5dT/GGTAGCTCGGTCGGGGTGGGTGGGTTGGCAAGTCTGAT
miR-192-RBS-MET-H-BHQ1	CAATTCATAGGACAGCCGAGCTACCA(BHQ1)
miR-192-RBS-MET-S	CACCCACCCCGCTGACCTATGAATTGACAGCC
miR-192-mimic	GGCTGTCAATTCATAGGTCAG
miR-122-RBS-FGFR-E-Cy5	GCCGCG/iCy5dT/CTTTATGGCTGGGGATGGTGTGGGTTGCGGC
miR-122-RBS-FGFR-H-BHQ1	TGAGAATGGTGTTTGCCAGCCATAAAG(BHQ1)
miR-122-RBS-FGFR-S	ACCCACACCATCCAAACACCATTGTCACACTCCA
H-11	TGAGAATGGTGTTTGCCGAGCTACCA
S-11	CACCCACCCCGCAAACACCATTGTCACACTCCA

Table S3. Oligonucleotide sequences for functional validation in *in vivo* imaging

Primer Name	Sequence (5'---3')
miR-122-RBS-MET-E-Cy7	ATCAGGCTGGA/iCy7dT/GGTAGCTCGGTCGGGGTGGGTGGGTTG GCAAGTCTGAT
miR-122-RBS-MET-D-BHQ3	TGAGAATGGTGTTTGCCGAGCTACCA（BHQ3）
miR-122-RBS-MET-S	CACCCACCCCGCAAACACCATTGTCACACTCCA

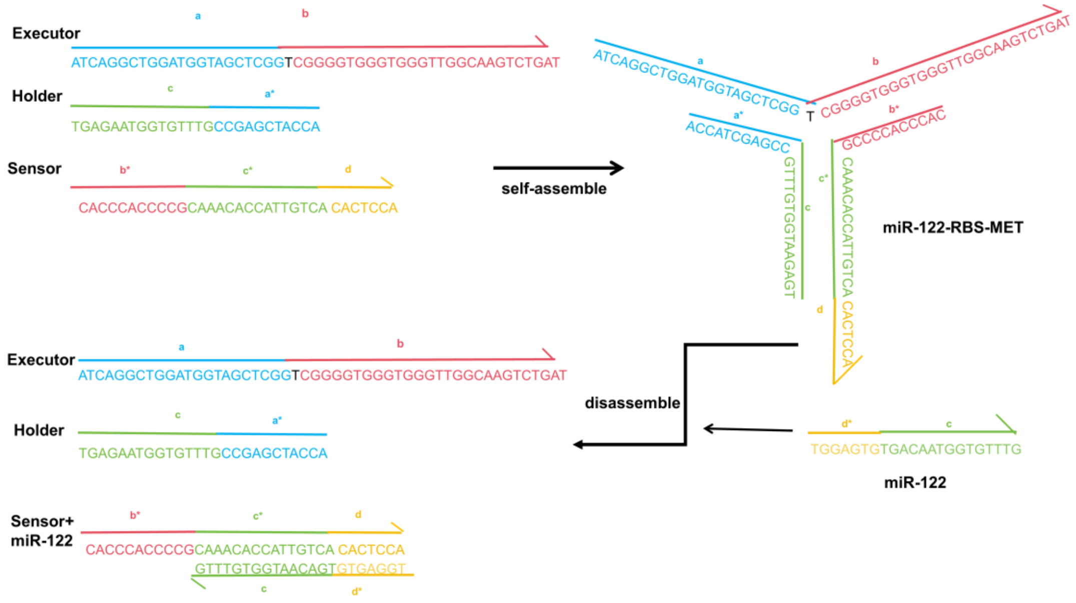

Figure S1. The schematic representation of miR-122-RBS-MET with detailed sequences.

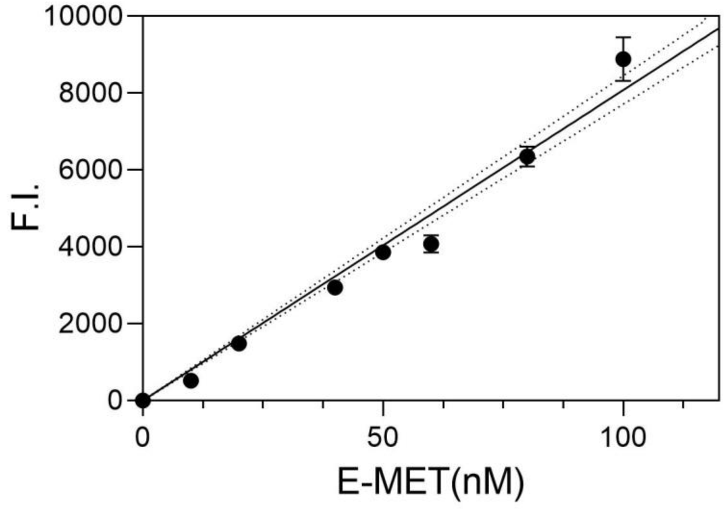

Figure S2. Linear fitting between the initial fluorescence intensity (F.I.) and the concentration of the Executor module.

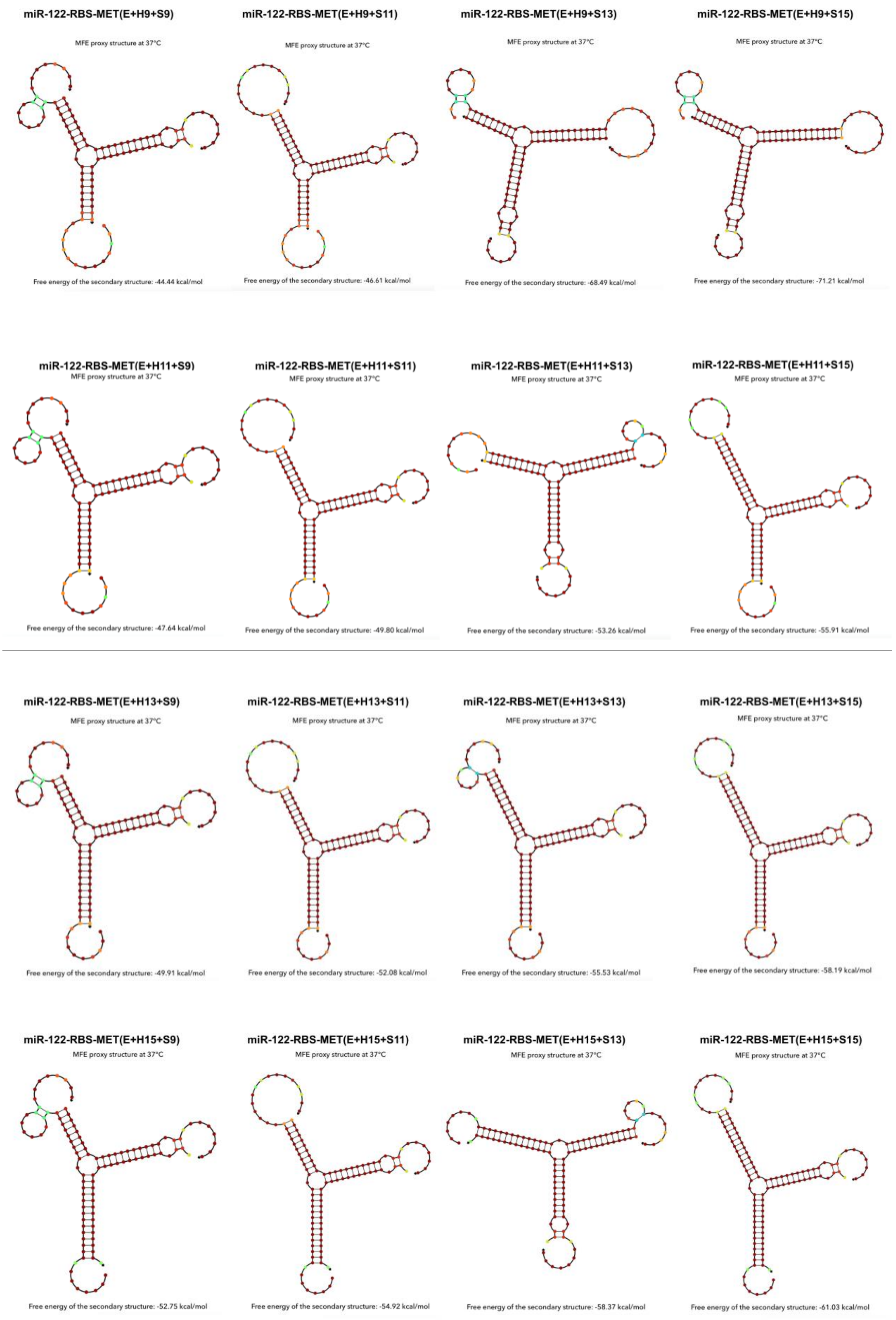

Figure S3. Secondary structure prediction of miR-RBS assembled in altered regulatory regions using the NUPACK software.

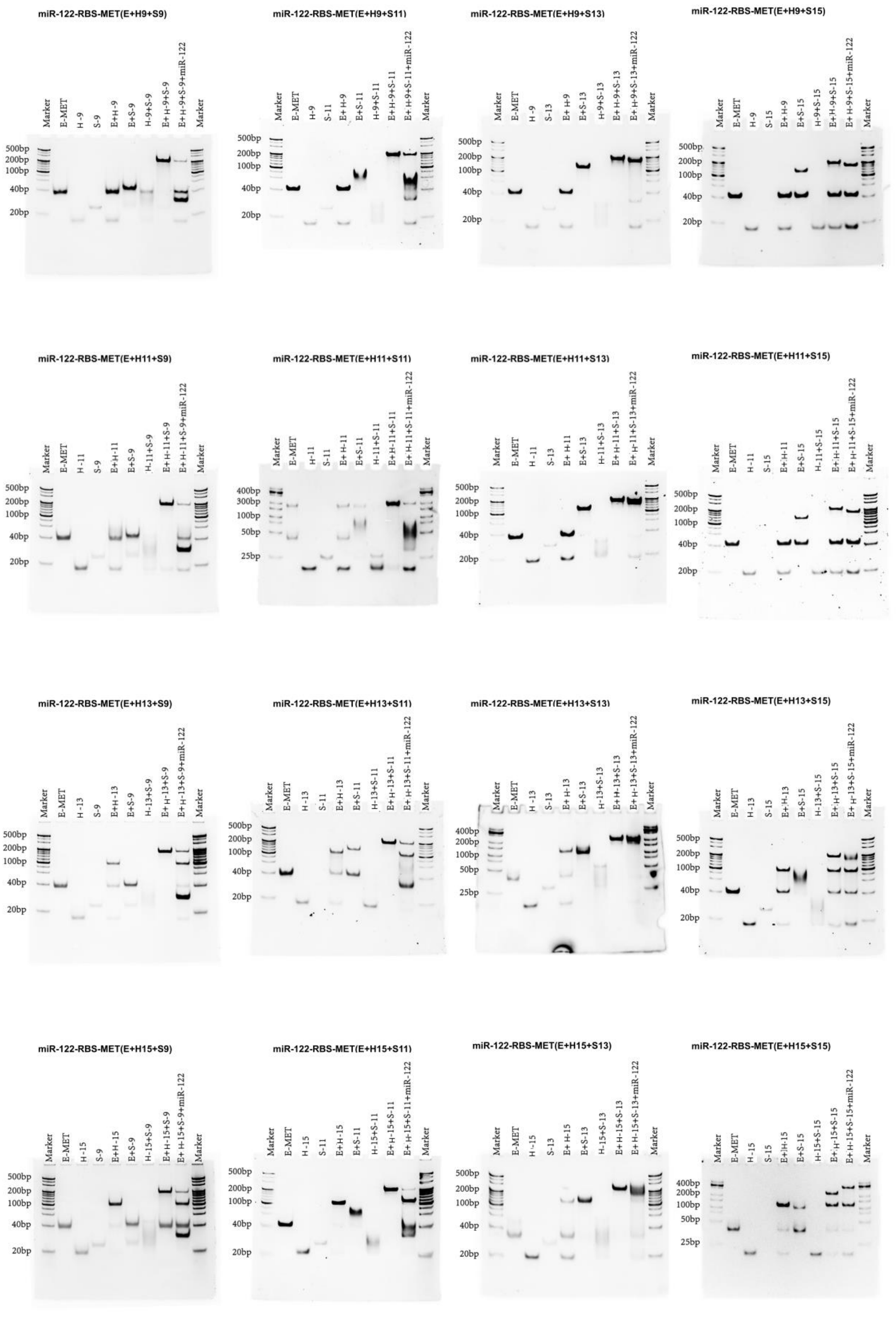

Figure S4. Validation of the assembly of regulatable miR-RBS and disassembly in response to miR-122 using PAGE

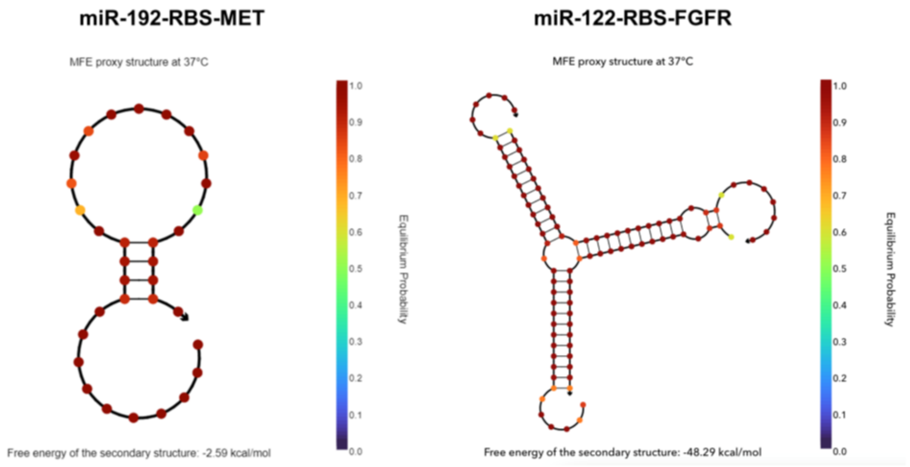

Figure S5. Secondary structure prediction of miR-RBS modularity using the NUPACK software.

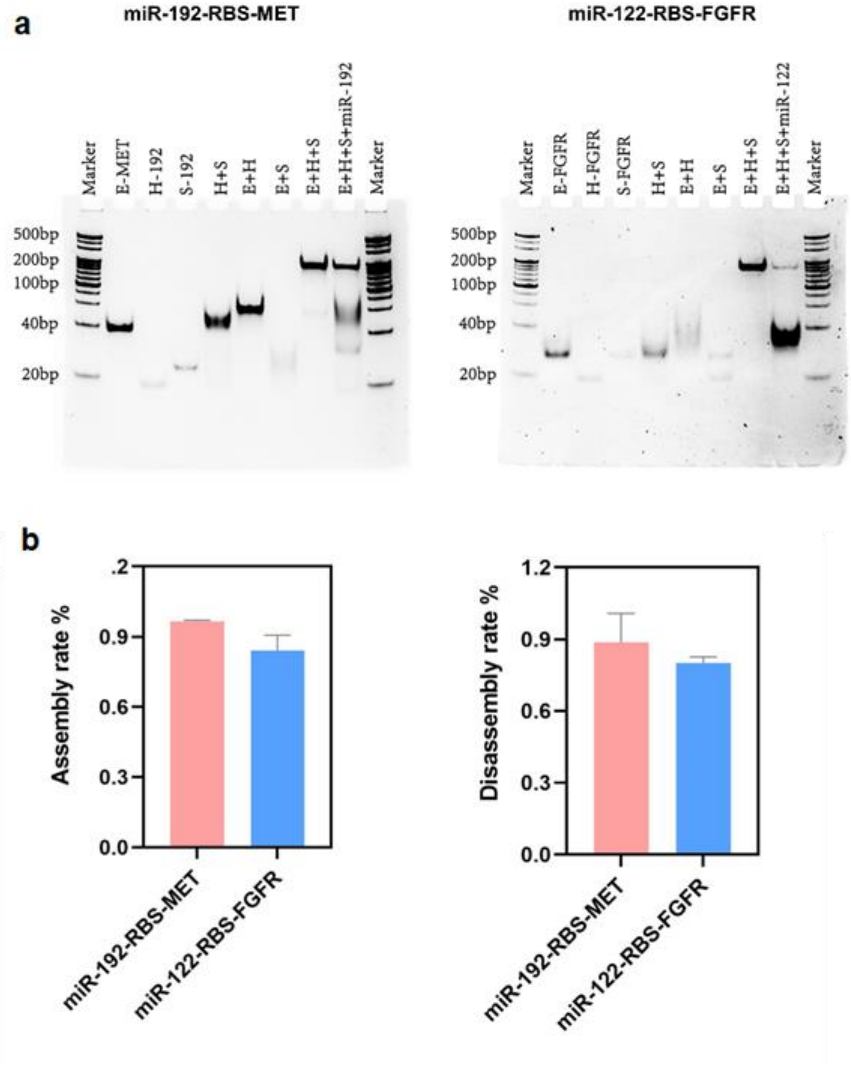

Figure S6. Validation of the miR-RBS assembly after modularization and response disassembly. (a) Gel electrophoresis analysis of the miR-192-RBS-MET and miR-122-RBS-FGFR assemblies. The gels show the successful assembly and disassembly of the miR-RBS complexes. PAGE was used to separate and visualize the different components. (b) Quantification of the assembly and disassembly rates of miR-RBS complexes. The gray values of each band from the gels (a) were quantified using Image J software, and the miR-RBS assembly and disassembly rates were calculated. Data are represented as mean ± S.D. (n = 3). Error bars represent standard deviation.
